# Supplementary material for: Facilitating Imaging Mass Spectrometry of Microbial Specialized Metabolites with METASPACE
Source: Metabolites. 2021 Jul 23;11(8):477. doi: 10.3390/metabo11080477 (PMC8401310; doi:10.3390/metabo11080477)

Supplementary Information

Facilitating imaging mass spectrometry of microbial specialized metabolites with METASPACE.

Don D. Nguyen[1], Veronika Saharuka[1], Vitaly Kovalev[1], Lachlan Stuart[1], Massimo Del Prete[2], Kinga M. Lubowiecka[2], René De Mot[3], Vittorio Venturi[4], Theodore Alexandrov[1, 5, 6, 7]

1 Structural and Computational Biology Unit, European Molecular Biology Laboratory, Heidelberg, Germany
2 Photolab, European Molecular Biology Laboratory, Heidelberg, Germany
3 Centre of Microbial and Plant Genetics, KU Leuven, Leuven, Belgium
4 International Centre for Genetic Engineering and Biotechnology, Trieste, Italy
5 Metabolomics Core Facility, European Molecular Biology Laboratory, Heidelberg, Germany
6 Molecular Medicine Partnership Unit (MMPU), European Molecular Biology Laboratory, Heidelberg, Germany
7 Skaggs School of Pharmacy and Pharmaceutical Sciences, University of California San Diego, La Jolla, CA, USA

This file contains Supplementary Tables 1-4 and Supplementary Figures 1-2.

Supplementary Table S1. Bacterial strains used for evaluation of microbial imaging mass spectrometry (imaging MS) with METASPACE.

Class	Genus and species	Strain Number
Actinobacteria	*Rhodococcus erythropolis*	43066
Actinobacteria	*Rhodococcus opacus*	43205
Actinobacteria	*Streptomyces coelicolor*	A2(3) / M145
Actinobacteria	*Streptomyces griseus subsp griseus*	40236
Actinobacteria	*Streptomyces mobaraensis*	
Actinobacteria	*Streptomyces lividans*	PM66
Actinobacteria	*Streptomyces roseolilacinus*	40173
Gamma Proteobacteria	*Pseudomonas savastanoi*	722
Gamma Proteobacteria	*Pseudomonas putida*	RW10S2
Bacilli	*Bacillus subtilis BGA*	618

Supplementary Table S2. Number of metabolites and unique molecular formulas from the Natural Products Atlas (NPA) com-pared to the number METASPACE annotations at various FDR percentages, with and without off-sample filtering.

		Total number of METASPACE annotations using NPA (FDR)							
		No filtering				Off-sample filtering			
**Metabolites in NPA**	**Unique molecular formulas in NPA**	**50%**	**20%**	**10%**	**5%**	**50%**	**20%**	**10%**	**5%**
5018	3727	1968	95	29	18	1045	64	20	12

Supplementary Table S3. Number of metabolites and unique molecular formulas from NPA after filtering for bacterial genus and species as compared to the number of unique molecular formulas annotated by METASPACE. Unique molecular formulas annotated by METASPACE are broken down by FDR 50% vs 20% and whether off-sample filtering was used.

				Unique molecular formulas annotated by METASPACE				
**Genus**	**species**	**Metabolites in NPA**	**Unique molecular formulas in NPA**	**FDR 50% no filter**	**FDR 50% off-sample filter**	**FDR 20% no filter**	**FDR 20% off-sample filter**	**Curated filtering**
Bacillus	–	389	275	40	26	2	2	9
	subtilis	101	81	8	5	2	2	9
Pseudomonas	–	266	235	39	27	2	1	10
	putida	3	2	0	0	0	0	9
	savastanoi	2	2	0	0	0	0	1
Rhodococcus	–	20	16	4	1	0	0	0
	erythropolis	3	3	1	0	0	0	0
	opacus	0	0	0	0	0	0	0
Streptomyces	–	4343	3201	512	285	30	22	34
	coelicolor	25	23	8	7	1	1	3
	griseus	106	89	24	15	0	0	3
	lividans	9	9	2	1	0	0	1
	mobaraensis	1	1	0	0	0	0	27
	roseolilacinus	0	0	0	0	0	0	0

Supplementary Table S4. 53 curated annotations from Figure 2 and Supplementary Figure 1 with direct URL to the annotation in METASPACE. Ordering is based on decreasing MSM score.

**FDR**	**MSM**	**Formula**	**Adduct**	***m/z***	**Molecule Names**	URL
5%	0.974	C54H95N9O16	+Na	1148.679	['WLIP; Viscosin; Massetolide F', 'Massetolide F', 'Viscosin']	https://metaspace2020.eu/annotations?db=NPA-2019-08&ds=2020-01-07_11h15m21s&mol=C54H95N9O16&add=%2BNa&fdr=0.5&sections=3&sort=formula
5%	0.927	C25H37NO4	+H	416.2795	['Trichobotrysin C', 'Piericidin A1', 'Phomacin C', 'Trichobotrysin B']	https://metaspace2020.eu/annotations?db=NPA-2019-08&ds=2020-01-07_11h15m21s&mol=C25H37NO4&add=%2BH&fdr=0.5&sections=3&sort=formula
5%	0.902	C51H80N12O14	+Na	1107.581	['Iturin A-8', 'Mycosubtilin D', 'Iturin A9']	https://metaspace2020.eu/annotations?db=NPA-2019-08&ds=2020-01-07_11h15m21s&mol=C51H80N12O14&add=%2BNa&fdr=0.5&sections=3&sort=formula
5%	0.872	C54H95N9O16	+K	1164.653	['WLIP; Viscosin; Massetolide F', 'Massetolide F', 'Viscosin']	https://metaspace2020.eu/annotations?db=NPA-2019-08&ds=2020-01-07_11h15m21s&mol=C54H95N9O16&add=%2BK&fdr=0.5&sections=3&sort=formula
5%	0.848	C51H80N12O14	+K	1123.555	['Iturin A-8', 'Mycosubtilin D', 'Iturin A9']	https://metaspace2020.eu/annotations?db=NPA-2019-08&ds=2020-01-07_11h15m21s&mol=C51H80N12O14&add=%2BK&fdr=0.5&sections=3&sort=formula
20%	0.777	C25H37NO4	+Na	438.2614	['Trichobotrysin C', 'Piericidin A1', 'Phomacin C', 'Trichobotrysin B']	https://metaspace2020.eu/annotations?db=NPA-2019-08&ds=2020-01-07_11h15m21s&mol=C25H37NO4&add=%2BNa&fdr=0.5&sections=3&sort=formula
20%	0.733	C53H93N7O13	+Na	1058.672	['[Ile7]-surfactin', 'Pumilacidin B', 'Leu7-Surfactin', 'Not named', 'Not named', 'Not named', 'Gageopeptin A']	https://metaspace2020.eu/annotations?db=NPA-2019-08&ds=2020-01-07_11h15m21s&mol=C53H93N7O13&add=%2BNa&fdr=0.5&sections=3&sort=formula
50%	0.729	C53H93N9O16	+K	1150.637	['Massetolide E', 'Massetolide E']	https://metaspace2020.eu/annotations?db=NPA-2019-08&ds=2020-01-07_11h15m21s&mol=C53H93N9O16&add=%2BK&fdr=0.5&sections=3&sort=formula
50%	0.717	C25H37NO4	+K	454.2354	['Trichobotrysin C', 'Piericidin A1', 'Phomacin C', 'Trichobotrysin B']	https://metaspace2020.eu/annotations?db=NPA-2019-08&ds=2020-01-07_11h15m21s&mol=C25H37NO4&add=%2BK&fdr=0.5&sections=3&sort=formula
50%	0.619	C53H93N7O13	+K	1074.646	['[Ile7]-surfactin', 'Pumilacidin B', 'Leu7-Surfactin', 'Not named', 'Not named', 'Not named', 'Gageopeptin A']	https://metaspace2020.eu/annotations?db=NPA-2019-08&ds=2020-01-07_11h15m21s&mol=C53H93N7O13&add=%2BK&fdr=0.5&sections=3&sort=formula
50%	0.611	C25H33N3O	+H	392.2696	['Butylcyclohexylprodigiosin', 'Metacycloprodigiosin', 'Methyl-cyclodecylprodiginine', 'Propyl-metacyclooctylprodiginine']	https://metaspace2020.eu/annotations?db=NPA-2019-08&ds=2020-01-07_11h15m21s&mol=C25H33N3O&add=%2BH&fdr=0.5&sections=3&sort=formula
50%	0.594	C25H35N3O	+H	394.2852	['Undecylprodigiosin', 'Prodigiosin 25-C']	https://metaspace2020.eu/annotations?db=NPA-2019-08&ds=2020-01-07_11h15m21s&mol=C25H35N3O&add=%2BH&fdr=0.5&sections=3&sort=formula

Supplementary Table S4 continued.

FDR	MSM	Formula	Adduct	*m/z*	Molecule Names
50%	0.541	$C_{14}H_{19}NO_4$	+H	266.1386	['3097-B2', 'Salinosporamide D', 'Fuzanin B', 'Fuzanin H', 'Salinosporamide G', 'Fuzanin A', 'Anisomycin']
https://metaspace2020.eu/annotations?db=NPA-2019-08&ds=2020-01-07_11h15m21s&mol=C14H19NO4&add=%2BH&fdr=0.5&sections=3&sort=formula					
20%	0.459	$C_{53}H_{93}N_9O_{16}$	+Na	1134.663	['Massetolide E', 'Massetolide E']
https://metaspace2020.eu/annotations?db=NPA-2019-08&ds=2020-01-07_11h15m21s&mol=C53H93N9O16&add=%2BNa&fdr=0.5&sections=3&sort=formula					
50%	0.438	$C_{28}H_{36}N_4O_6$	+Na	547.2527	['Safracin A']
https://metaspace2020.eu/annotations?db=NPA-2019-08&ds=2020-01-07_11h15m21s&mol=C28H36N4O6&add=%2BNa&fdr=0.5&sections=3&sort=formula					
50%	0.277	$C_{14}H_{19}NO_4$	+Na	288.1206	['3097-B2', 'Salinosporamide D', 'Fuzanin B', 'Fuzanin H', 'Salinosporamide G', 'Fuzanin A', 'Anisomycin']
https://metaspace2020.eu/annotations?db=NPA-2019-08&ds=2020-01-07_11h15m21s&mol=C14H19NO4&add=%2BNa&fdr=0.5&sections=3&sort=formula					
50%	0.188	$C_{14}H_{19}NO_4$	+K	304.0945	['3097-B2', 'Salinosporamide D', 'Fuzanin B', 'Fuzanin H', 'Salinosporamide G', 'Fuzanin A', 'Anisomycin']
https://metaspace2020.eu/annotations?db=NPA-2019-08&ds=2020-01-07_11h15m21s&mol=C14H19NO4&add=%2BK&fdr=0.5&sections=3&sort=formula					
50%	0.185	$C_{18}H_{36}N_6O_5$	+H	417.282	["2"-N-formimidoylistamycin B"]
https://metaspace2020.eu/annotations?db=NPA-2019-08&ds=2020-01-07_11h15m21s&mol=C18H36N6O5&add=%2BH&fdr=0.5&sections=3&sort=formula					
50%	0.169	$C_{54}H_{95}N_9O_{16}$	+H	1126.697	['WLIP; Viscosin; Massetolide F', 'Massetolide F', 'Viscosin']
https://metaspace2020.eu/annotations?db=NPA-2019-08&ds=2020-01-07_11h15m21s&mol=C54H95N9O16&add=%2BH&fdr=0.5&sections=3&sort=formula					
50%	0.14	$C_{56}H_{99}N_9O_{16}$	+Na	1176.71	['Massetolide B/H', 'Massetolide H', 'Massetolide B']
https://metaspace2020.eu/annotations?db=NPA-2019-08&ds=2020-01-07_11h15m21s&mol=C56H99N9O16&add=%2BNa&fdr=0.5&sections=3&sort=formula					
50%	0.135	$C_{25}H_{37}NO_3$	+Na	422.2665	['Trichobotrysin D', 'Piericidin F']
https://metaspace2020.eu/annotations?db=NPA-2019-08&ds=2020-01-07_11h15m21s&mol=C25H37NO3&add=%2BNa&fdr=0.5&sections=3&sort=formula					
50%	0.126	$C_{25}H_{37}NO_3$	+H	400.2846	['Trichobotrysin D', 'Piericidin F']
https://metaspace2020.eu/annotations?db=NPA-2019-08&ds=2020-01-07_11h15m21s&mol=C25H37NO3&add=%2BH&fdr=0.5&sections=3&sort=formula					
50%	0.125	$C_{34}H_{46}N_2O_8$	+K	649.2885	['UCF-116-C', 'Ansatrienin A3', 'Ansatrienin A2']
https://metaspace2020.eu/annotations?db=NPA-2019-08&ds=2020-01-07_11h15m21s&mol=C34H46N2O8&add=%2BK&fdr=0.5&sections=3&sort=formula					
50%	0.124	$C_{20}H_{27}NO_4$	+H	346.2012	['Radianspene J', "(2E)-11-(4'-aminophenyl)-5,9-O-cyclo-4,6,8-trimethyl-11-oxo-undec-2-enoic acid"]
https://metaspace2020.eu/annotations?db=NPA-2019-08&ds=2020-01-07_11h15m21s&mol=C20H27NO4&add=%2BH&fdr=0.5&sections=3&sort=formula					

Supplementary Table S4 continued.

FDR	MSM	Formula	Adduct	*m/z*	Molecule Names
50%	0.122	C52H91N7O13	+Na	1044.657	['Not named', '[Ile4]Surfactin', '[Leu4]Surfactin', 'Not named', '[Leu-7]surfactin', '[Ile2,4,7]Surfactin', 'Not named', 'Surfactin', 'Gageopeptin B']

https://metaspace2020.eu/annotations?db=NPA-2019-08&ds=2020-01-07_11h15m21s&mol=C52H91N7O13&add=%2BNa&fdr=0.5&sections=3&sort=formula

FDR	MSM	Formula	Adduct	*m/z*	Molecule Names
50%	0.121	C56H99N9O16	+K	1192.684	['Massetolide B/H', 'Massetolide H', 'Massetolide B']

https://metaspace2020.eu/annotations?db=NPA-2019-08&ds=2020-01-07_11h15m21s&mol=C56H99N9O16&add=%2BK&fdr=0.5&sections=3&sort=formula

FDR	MSM	Formula	Adduct	*m/z*	Molecule Names
50%	0.119	C10H13NO4	+H	212.0917	['Farinomalein', 'N-salicyloyl-2-aminopropan-1,3-diol', 'Pestalactam B', '2,5-Dihydroxy-4-methoxymethyl-acetanilide', 'Makomotine D', 'CJ-14,877']

https://metaspace2020.eu/annotations?db=NPA-2019-08&ds=2020-01-07_11h15m21s&mol=C10H13NO4&add=%2BH&fdr=0.5&sections=3&sort=formula

FDR	MSM	Formula	Adduct	*m/z*	Molecule Names
50%	0.118	C40H70O8	+K	717.4702	['Campechic acid A']

https://metaspace2020.eu/annotations?db=NPA-2019-08&ds=2020-01-07_11h15m21s&mol=C40H70O8&add=%2BK&fdr=0.5&sections=3&sort=formula

FDR	MSM	Formula	Adduct	*m/z*	Molecule Names
50%	0.118	C55H97N9O16	+Na	1162.695	['Pseudophomin A; Massetolide A/D/G', 'Massetolide A', 'Massetolide D', 'Massetolide G', 'Pseudophomin A']

https://metaspace2020.eu/annotations?db=NPA-2019-08&ds=2020-01-07_11h15m21s&mol=C55H97N9O16&add=%2BNa&fdr=0.5&sections=3&sort=formula

FDR	MSM	Formula	Adduct	*m/z*	Molecule Names
50%	0.113	C27H35NO4	+H	438.2638	['Heronamide D']

https://metaspace2020.eu/annotations?db=NPA-2019-08&ds=2020-01-07_11h15m21s&mol=C27H35NO4&add=%2BH&fdr=0.5&sections=3&sort=formula

FDR	MSM	Formula	Adduct	*m/z*	Molecule Names
50%	0.111	C55H97N9O16	+K	1178.668	['Pseudophomin A; Massetolide A/D/G', 'Massetolide A', 'Massetolide D', 'Massetolide G', 'Pseudophomin A']

https://metaspace2020.eu/annotations?db=NPA-2019-08&ds=2020-01-07_11h15m21s&mol=C55H97N9O16&add=%2BK&fdr=0.5&sections=3&sort=formula

FDR	MSM	Formula	Adduct	*m/z*	Molecule Names
50%	0.106	C24H35NO3	+Na	408.2509	['Ascosalipyrrolidinone B', 'TMC-169', 'Mer-A2026B']

https://metaspace2020.eu/annotations?db=NPA-2019-08&ds=2020-01-07_11h15m21s&mol=C24H35NO3&add=%2BNa&fdr=0.5&sections=3&sort=formula

FDR	MSM	Formula	Adduct	*m/z*	Molecule Names
50%	0.097	C50H78N12O14	+Na	1093.565	['Iturin A-7', 'Iturin A6']

https://metaspace2020.eu/annotations?db=NPA-2019-08&ds=2020-01-07_11h15m21s&mol=C50H78N12O14&add=%2BNa&fdr=0.5&sections=3&sort=formula

FDR	MSM	Formula	Adduct	*m/z*	Molecule Names
50%	0.093	C13H16N2O3	+H	249.1233	['Salinazinone B', 'Abbeymycin', 'Spinoxazine B']

https://metaspace2020.eu/annotations?db=NPA-2019-08&ds=2020-01-07_11h15m21s&mol=C13H16N2O3&add=%2BH&fdr=0.5&sections=3&sort=formula

FDR	MSM	Formula	Adduct	*m/z*	Molecule Names
50%	0.089	C24H35NO3	+H	386.2689	['Ascosalipyrrolidinone B', 'TMC-169', 'Mer-A2026B']

https://metaspace2020.eu/annotations?db=NPA-2019-08&ds=2020-01-07_11h15m21s&mol=C24H35NO3&add=%2BH&fdr=0.5&sections=3&sort=formula

FDR	MSM	Formula	Adduct	*m/z*	Molecule Names
50%	0.087	C43H51NO16	+K	876.2839	['Sulfurmycin B']

https://metaspace2020.eu/annotations?db=NPA-2019-08&ds=2020-01-07_11h15m21s&mol=C43H51NO16&add=%2BK&fdr=0.5&sections=3&sort=formula

Supplementary Table S4 continued.

FDR	MSM	Formula	Adduct	*m/z*	Molecule Names
50%	0.079	C24H35NO4	+Na	424.2458	['Aspochalasin J', 'SN-I98-D', 'L-755807', 'Gymnastatin H', 'Aspergillin PZ', 'Aspochalasin P', 'Aspochalasin D', 'Trichalasin H', 'Aspochalasin M', 'Aspochalasin S', 'Piericidin E1']
https://metaspace2020.eu/annotations?db=NPA-2019-08&ds=2020-01-07_11h15m21s&mol=C24H35NO4&add=%2BNa&fdr=0.5&sections=3&sort=formula					
50%	0.079	C24H35NO3	+K	424.2248	['Ascosalipyrrolidinone B', 'TMC-169', 'Mer-A2026B']
https://metaspace2020.eu/annotations?db=NPA-2019-08&ds=2020-01-07_11h15m21s&mol=C24H35NO3&add=%2BK&fdr=0.5&sections=3&sort=formula					
50%	0.071	C23H33NO5	+H	404.2431	['Salternamide B', 'Neopestalotin C', "12'-hydroxy gymnastatin N", 'Chartarutine A']
https://metaspace2020.eu/annotations?db=NPA-2019-08&ds=2020-01-07_11h15m21s&mol=C23H33NO5&add=%2BH&fdr=0.5&sections=3&sort=formula					
50%	0.064	C23H33NO5	+Na	426.2251	['Salternamide B', 'Neopestalotin C', "12'-hydroxy gymnastatin N", 'Chartarutine A']
https://metaspace2020.eu/annotations?db=NPA-2019-08&ds=2020-01-07_11h15m21s&mol=C23H33NO5&add=%2BNa&fdr=0.5&sections=3&sort=formula					
50%	0.063	C20H29NO5	+H	364.2118	['(2E)-11-(4-aminophenyl)-5,9-dihydroxy-4,6,8-trimethyl-11-oxo-undec-2-enoic acid']
https://metaspace2020.eu/annotations?db=NPA-2019-08&ds=2020-01-07_11h15m21s&mol=C20H29NO5&add=%2BH&fdr=0.5&sections=3&sort=formula					
50%	0.062	C18H36N6O5	+K	455.2378	["2"-N-formimidoylistamycin B"]
https://metaspace2020.eu/annotations?db=NPA-2019-08&ds=2020-01-07_11h15m21s&mol=C18H36N6O5&add=%2BK&fdr=0.5&sections=3&sort=formula					
50%	0.059	C26H39NO3	+Na	436.2822	['Termitomycamide A', 'Trichobotrysin E', 'Myceliothermophin B', 'Myceliothermophin A', 'Mer-A2026A']
https://metaspace2020.eu/annotations?db=NPA-2019-08&ds=2020-01-07_11h15m21s&mol=C26H39NO3&add=%2BNa&fdr=0.5&sections=3&sort=formula					
50%	0.057	C29H54N10O12	+Na	757.3814	['Tsukubachelin B']
https://metaspace2020.eu/annotations?db=NPA-2019-08&ds=2020-01-07_11h15m21s&mol=C29H54N10O12&add=%2BNa&fdr=0.5&sections=3&sort=formula					
50%	0.057	C50H77N11O15	+Na	1094.549	['Iturin C3']
https://metaspace2020.eu/annotations?db=NPA-2019-08&ds=2020-01-07_11h15m21s&mol=C50H77N11O15&add=%2BNa&fdr=0.5&sections=3&sort=formula					
50%	0.055	C25H37NO3	+K	438.2405	['Trichobotrysin D', 'Piericidin F']
https://metaspace2020.eu/annotations?db=NPA-2019-08&ds=2020-01-07_11h15m21s&mol=C25H37NO3&add=%2BK&fdr=0.5&sections=3&sort=formula					
50%	0.036	C50H78N12O14	+K	1109.539	['Iturin A-7', 'Iturin A6']
https://metaspace2020.eu/annotations?db=NPA-2019-08&ds=2020-01-07_11h15m21s&mol=C50H78N12O14&add=%2BK&fdr=0.5&sections=3&sort=formula					
50%	0.028	C52H91N7O13	+K	1060.631	['Not named', '[Ile4]Surfactin', '[Leu4]Surfactin', 'Not named', '[Leu-7]surfactin', '[Ile2,4,7]Surfactin', 'Not named', 'Surfactin', 'Gageopeptin B']
https://metaspace2020.eu/annotations?db=NPA-2019-08&ds=2020-01-07_11h15m21s&mol=C52H91N7O13&add=%2BK&fdr=0.5&sections=3&sort=formula					

Supplementary Table S4 continued.

FDR	MSM	Formula	Adduct	*m/z*	Molecule Names
50%	0.025	C47H78O14	+Na	889.5283	['Endusamycin']
https://metaspace2020.eu/annotations?db=NPA-2019-08&ds=2020-01-07_11h15m21s&mol=C47H78O14&add=%2BNa&fdr=0.5&sections=3&sort=formula					
50%	0.022	C23H42O7	+K	469.2562	['8-Deoxylankolide']
https://metaspace2020.eu/annotations?db=NPA-2019-08&ds=2020-01-07_11h15m21s&mol=C23H42O7&add=%2BK&fdr=0.5&sections=3&sort=formula					
50%	0.02	C20H27NO4	+Na	368.1832	['Radianspene J', "(2E)-11-(4'-aminophenyl)-5,9-O-cyclo-4,6,8-trimethyl-11-oxo-undec-2-enoic acid"]
https://metaspace2020.eu/annotations?db=NPA-2019-08&ds=2020-01-07_11h15m21s&mol=C20H27NO4&add=%2BNa&fdr=0.5&sections=3&sort=formula					
50%	0.013	C17H29N7O8	+K	498.1709	['Glycinothricin']
https://metaspace2020.eu/annotations?db=NPA-2019-08&ds=2020-01-07_11h15m21s&mol=C17H29N7O8&add=%2BK&fdr=0.5&sections=3&sort=formula					
50%	0.013	C20H27NO4	+K	384.1571	['Radianspene J', "(2E)-11-(4'-aminophenyl)-5,9-O-cyclo-4,6,8-trimethyl-11-oxo-undec-2-enoic acid"]
https://metaspace2020.eu/annotations?db=NPA-2019-08&ds=2020-01-07_11h15m21s&mol=C20H27NO4&add=%2BK&fdr=0.5&sections=3&sort=formula					

Supplementary Figure S1. Imaging MS and annotated molecules by METASPACE using NPA. All annotations filtered by or-ganism type, genus, and then hand selected for reasonable ion distribution, resulting in the 13th to 53rd highest ranked an-notations. Optical image of *Streptomyces griseus subsp griseus* 40236, *Bacillus subtilis* BGA 618, *Pseudomonas savastanoi* 722, *Streptomyces coelicolor* A2(3) / M145, *Streptomyces lividans* PM66, *Rhodococcus erythropolis* 43066, *Rhodococcus opacus* 43205, *Streptomyces roseolilacinus* 40173, *Streptomyces mobaraensis*, *Pseudomonas putida* RW10S2, from left to right, top to bottom. Ion images are arranged from increasing to decreasing MSM score from left to right, top to bottom, while *m/z* 228 was selected as a background ion image to show the locations of all the bacteria. Each ion image is denoted by the adduct detected, molecule name, the monoisotopic *m/z* value, MSM score, and FDR percentage. Molecule names with an * represent annotations with potential analogs.

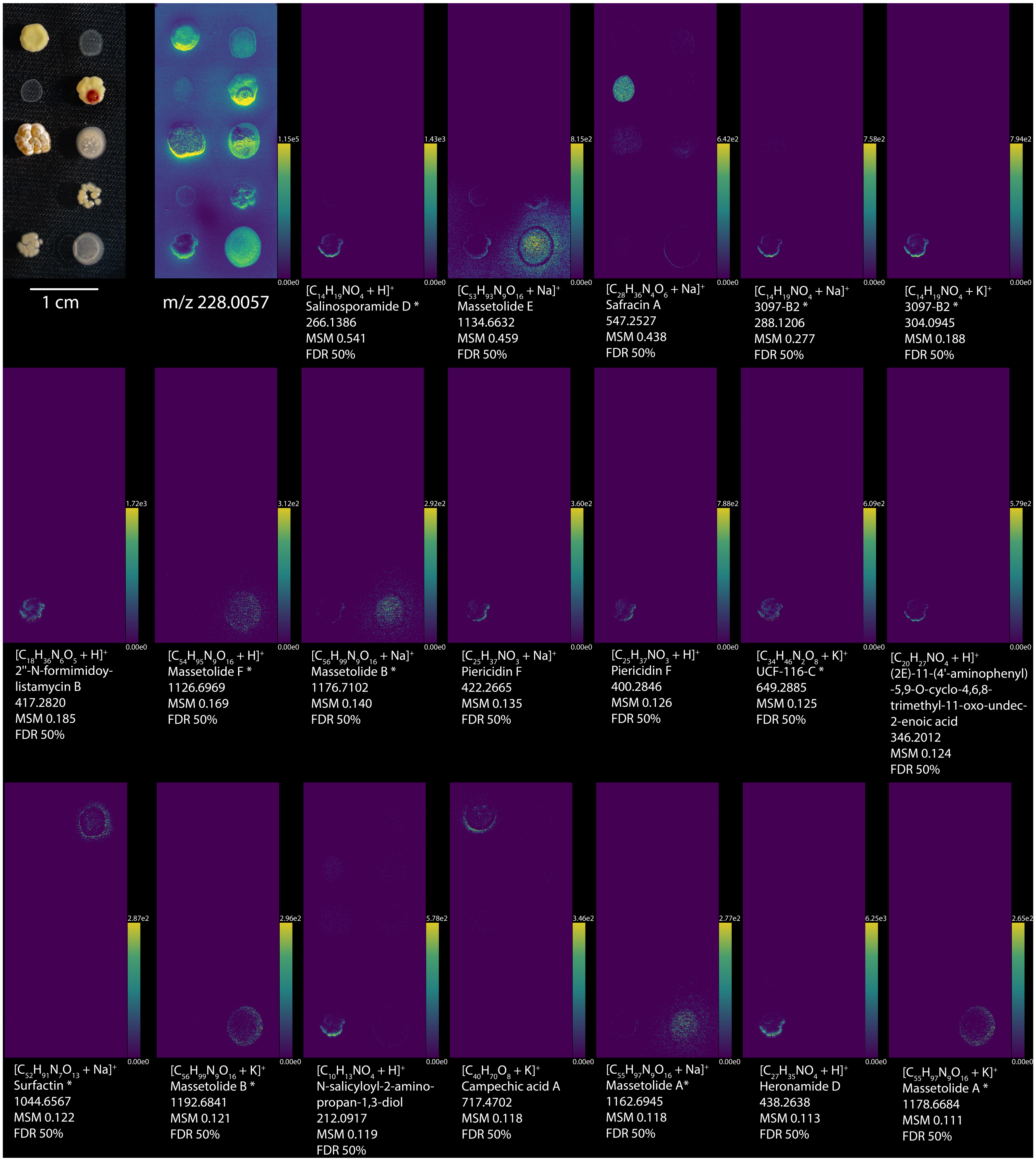

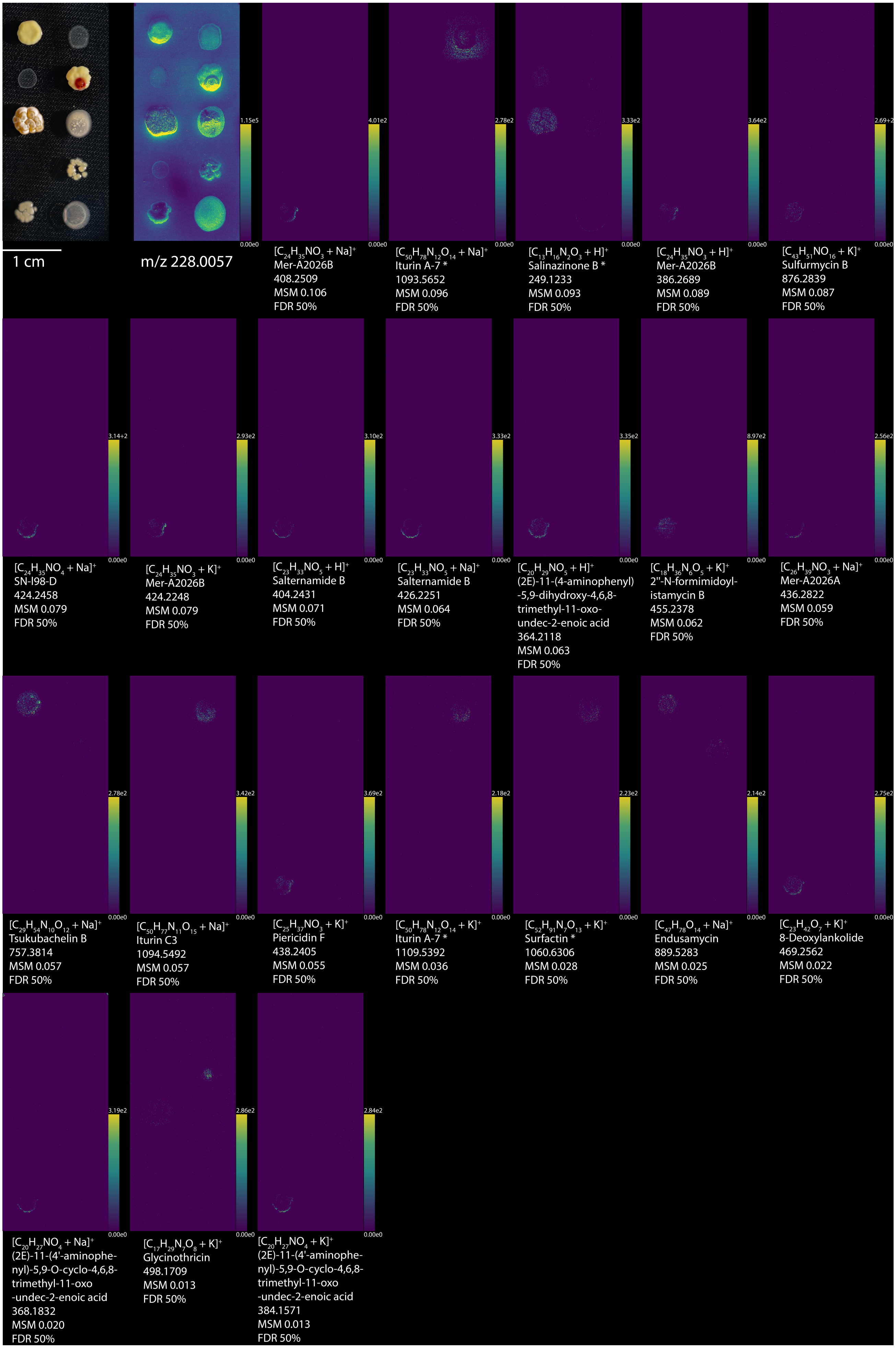
1 cm
m/z 228.0057
$[C_{24}H_{35}NO_3 + Na]^+$ Mer-A2026B 408.2509 MSM 0.106 FDR 50%
$[C_{50}H_{78}N_{12}O_{14} + Na]^+$ Iturin A-7 * 1093.5652 MSM 0.096 FDR 50%
$[C_{13}H_{16}N_2O_3 + H]^+$ Salinazinone B * 249.1233 MSM 0.093 FDR 50%
$[C_{24}H_{35}NO_3 + H]^+$ Mer-A2026B 386.2689 MSM 0.089 FDR 50%
$[C_{43}H_{51}NO_{16} + K]^+$ Sulfurmycin B 876.2839 MSM 0.087 FDR 50%
$[C_{24}H_{35}NO_4 + Na]^+$ SN-198-D 424.2458 MSM 0.079 FDR 50%
$[C_{24}H_{35}NO_3 + K]^+$ Mer-A2026B 424.2248 MSM 0.079 FDR 50%
$[C_{23}H_{33}NO_5 + H]^+$ Salternamide B 404.2431 MSM 0.071 FDR 50%
$[C_{23}H_{33}NO_5 + Na]^+$ Salternamide B 426.2251 MSM 0.064 FDR 50%
$[C_{20}H_{29}NO_5 + H]^+$ (2E)-11-(4-aminophenyl) -5,9-dihydroxy-4,6,8-trimethyl-11-oxo-undec-2-enoic acid 364.2118 MSM 0.063 FDR 50%
$[C_{18}H_{36}N_6O_5 + K]^+$ 2''-N-formimidoyl-istamycin B 455.2378 MSM 0.062 FDR 50%
$[C_{26}H_{39}NO_3 + Na]^+$ Mer-A2026A 436.2822 MSM 0.059 FDR 50%
$[C_{29}H_{54}N_{10}O_{12} + Na]^+$ Tsukubachelin B 757.3814 MSM 0.057 FDR 50%
$[C_{50}H_{77}N_{11}O_{15} + Na]^+$ Iturin C3 1094.5492 MSM 0.057 FDR 50%
$[C_{25}H_{37}NO_3 + K]^+$ Piericidin F 438.2405 MSM 0.055 FDR 50%
$[C_{50}H_{78}N_{12}O_{14} + K]^+$ Iturin A-7 * 1109.5392 MSM 0.036 FDR 50%
$[C_{52}H_{91}N_7O_{13} + K]^+$ Surfactin * 1060.6306 MSM 0.028 FDR 50%
$[C_{47}H_{78}O_{14} + Na]^+$ Endusamycin 889.5283 MSM 0.025 FDR 50%
$[C_{23}H_{42}O_7 + K]^+$ 8-Deoxylankolide 469.2562 MSM 0.022 FDR 50%
$[C_{20}H_{27}NO_4 + Na]^+$ (2E)-11-(4'-aminophe-nyl)-5,9-O-cyclo-4,6,8-trimethyl-11-oxo -undec-2-enoic acid 368.1832 MSM 0.020 FDR 50%
$[C_{17}H_{29}N_7O_8 + K]^+$ Glycinothricin 498.1709 MSM 0.013 FDR 50%
$[C_{20}H_{27}NO_4 + K]^+$ (2E)-11-(4'-aminophe-nyl)-5,9-O-cyclo-4,6,8-trimethyl-11-oxo -undec-2-enoic acid 384.1571 MSM 0.013 FDR 50%

Supplementary Figure S2. Average mass spectrum of the entire imaging area (outlined in pink) including all 10 microbes. The imzML file was loaded into MSiReader (v1.01) and the polygon tool was used to select the region of interest, followed by exporting and viewing the average mass spectrum from the selected pixels.

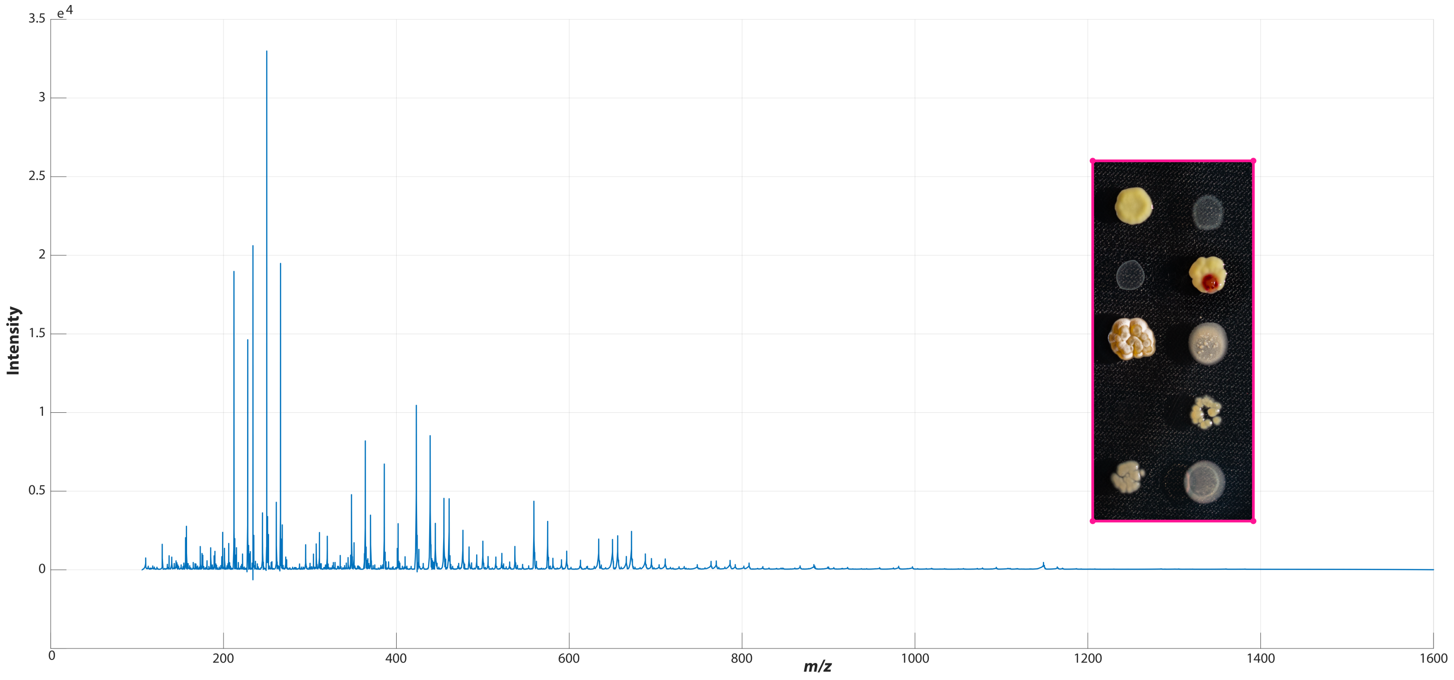

Supplement: Supplementary file 1 [file metabolites-11-00477-s001.zip › metabolites-1273287-supplementary.pdf]
